# Editorial: Advancing vocal biomarkers and voice AI in healthcare: multidisciplinary focus on responsible and effective development and use

**DOI:** 10.3389/fdgth.2026.1811486

**Published:** 2026-04-10

**Authors:** Jean-Christophe Bélisle-Pipon, Jamie Toghranegar, Maria E. Powell

**Affiliations:** 1 Faculty of Health Sciences, Simon Fraser University, Burnaby, BC, Canada; 2 Department of Otolaryngology Head & Neck Surgery, Morsani College of Medicine, University of South Florida, Tampa, FL, United States; 3 Department of Hearing & Speech Sciences, Vanderbilt University Medical Center, Nashville, TN, United States; 4 Department of Otolaryngology—Head and Neck Surgery, Vanderbilt University Medical Center, Nashville, TN, United States

**Keywords:** vocal biomarkers, voice AI, audiomics, clinical validation, implementation science, AI governance, digital biomarkers, standardization

The human voice is an extraordinary instrument. For millennia, it has been our primary tool for connection, culture, and communication. But to the ears of a clinician, it has always been something more: a subtle, analog indicator of underlying health. Just as the stethoscope revolutionized medicine by amplifying the internal sounds of the body, advances in artificial intelligence (AI) are now poised to translate the ancient art of listening into a precise, scalable, and data-driven science (1). Even without technology, a doctor can often hear the breathy strain of heart failure, the dampened affect of depression, or the motor deficits of a neurological condition long before a formal diagnosis is rendered. Today, machine learning (ML) promises to scale this observation, creating a future where a simple, non-invasive voice recording can aid in the screening, diagnosis, and monitoring of a vast spectrum of human diseases (2).

This promise is the driving force behind the rapid growth of voice AI in healthcare (3). However, the distance between technical possibility and clinical proof remains significant. The path from a promising algorithm to a trusted, effective, and equitable healthcare tool is fraught with profound challenges (4, 5). How do we prove that a subtle change in vocal jitter is strictly linked to heart failure? How do we build robust, billion-parameter models when data are collected from noisy, real-world environments? And crucially, how do we train algorithms that are fair across languages, dialects, and healthcare contexts while protecting privacy and avoiding the inequities embedded in earlier waves of digital health innovation?

This Research Topic, “Advancing Vocal Biomarkers and Voice AI in Healthcare: Multidisciplinary Focus on Responsible and Effective Development and Use,” was convened to confront these questions directly. Rather than celebrate novelty, it evaluates the field's foundations: measurement validity, implementation feasibility, and governance. The collection of articles herein does not offer simple answers. Instead, it provides a detailed, multidisciplinary snapshot of a field in its critical formative years. It brings together work from clinicians, data scientists, ethicists, engineers, and implementation specialists, charting a course that is not only technically innovative but clinically valid, ethically sourced, and socially responsible.

Voice AI has crossed from speculation to structured clinical inquiry. The thirteen papers in this collection demonstrate that voice can now function as a regulated physiological signal, provided its collection and interpretation are governed by standardization, usability, and trust. The contributions are presented here not as a simple list, but as a dialogue, organized around the four key pillars required to build this new frontier in digital health.

## 1 The foundation: rigorous clinical and scientific validation

Before any algorithm can be trusted, the fundamental link between voice and pathology must be rigorously established. Two articles in this collection provide exemplary models for this foundational work, moving from complex systemic disease to specific, localized pathology.

Kerwagen et al., in their paper “Vocal biomarkers in heart failure: design, rationale and baseline characteristics of the AHF-Voice study,” offer a masterclass in prospective clinical validation. They are not simply analyzing an existing, convenient dataset. They are meticulously building a new one. Their study protocol, which collects daily voice recordings via a smartphone app from patients hospitalized for acute heart failure (AHF), provides a crucial blueprint for the entire field. By correlating voice alterations with objective clinical markers of congestion (such as weight gain and edema), their work moves beyond “black box” correlation to uncover the potential pathophysiological mechanism (e.g., fluid overload affecting the vocal folds). The study's multi-dimensional design, integrating auditory, visual, clinician-rated, and patient-reported measures, sets a new standard for methodological completeness.

Pivoting from systemic disease to localized pathology, Jenkins et al. provide a focused exploratory analysis in “Voice as a biomarker: exploratory analysis for benign and malignant vocal fold lesions.” Using the initial release of the Bridge2AI-Voice dataset, their work tackles a classic diagnostic challenge: distinguishing laryngeal cancer and benign lesions from healthy voices. Their contribution lies in identifying which specific acoustic features hold the most diagnostic promise. They demonstrate that the harmonic-to-noise ratio (HNR), a measure of vocal clarity, is a potent differentiator. By anchoring acoustic markers in vocal-fold physiology, their results reinforce a principle emerging across this collection: mechanistically interpretable features yield the most clinically durable models.

## 2 The toolkit: building the technical backbone

With a clinical target in sight, the focus shifts to the technical “how.” How do we reliably capture and analyze voice data in the real world? This collection features articles that build the technical backbone for voice AI, from the physical device to the analytical software.

The ubiquitous smartphone is the field's greatest asset and its greatest liability. Awan et al., in “Influence of recording instrumentation on measurements of voice in sentence contexts: use of smartphones and tablets,” provide essential “ground truth” research. They systematically test how different consumer-grade devices, microphones (internal vs. headset), and background noise levels distort the acoustic measures used in diagnostics. Their finding, that measures like Cepstral Peak Prominence (CPP) are significantly affected by device and noise but remain highly correlated with the laboratory standard, is significant. It confirms that while raw values may differ, the diagnostic *signal* can be preserved, paving the way for developing standardized, hardware-agnostic models. Their results (e.g., >0.9 correlation across devices) now define the empirical boundary between permissible variation and measurement bias.

Building on this need for standardization, Nylén “On Acoustic Voice Quality Index measurement reliability in digital health applications” seeks to refine our understanding of an existing clinical standard. The Acoustic Voice Quality Index (AVQI) is widely used, but its implementation in digital apps has been inconsistent. Nylén's narrative review and empirical evaluation answer a fundamental question: how much speech is *enough*? His finding that reliability is achieved at 50 words (approximately 20 s), a sample longer than most current recommendations, represents a key contribution to technical rigor in Voice AI. The Nylen argues that this result should be treated as a *de facto* minimum for mobile data collection, ensuring that measurements are statistically stable before they are clinically trusted.

Additionnaly, Shirk et al. demonstrate the sheer power of new generation AI in “Leveraging large language models for automated detection of velopharyngeal dysfunction in patients with cleft palate.” This paper seeks to act as a technical paradigm shift. Where traditional ML required painstaking feature engineering, this team repurposed OpenAI's *Whisper*, a pre-trained speech-to-text model for a complex diagnostic task: detecting hypernasality. The model achieved 97 percent accuracy, dramatically outperforming traditional models. Importantly, their smallest model outperformed the largest, underscoring that diagnostic value in healthcare is linked not to computational scale but to contextual training. This contribution is significant as it suggests that the heavy lifting of acoustic feature extraction may already be solved by foundational models. This could help democratizing the development of highly accurate clinical tools and allowing researchers to focus on validation and implementation.

Finally, Yan et al. move from diagnostic analysis to *interaction* analysis, tackling the immense challenge of processing massive, qualitative, real-world conversational datasets. In “Understanding older people's voice interactions with smart voice assistants: a new modified rule-based natural language processing model with human input,” they confront the limitations of manual coding (which is slow, subjective, and prone to human fatigue) and standard dictionary-based tools, which fail to capture the nuances of evolving dialogues. Their contribution is a hybrid Modified Rule-based NLP (MR-NLP) model, where human-derived insights are used to establish and iteratively refine the rules for an automated system. Testing this on interaction data from older adults using Smart Voice Assistants, they demonstrated their model was not only exponentially more efficient (using only 9% of the time required for manual coding), but also more accurate. Their work provides a critical, reproducible “human-in-the-loop” framework, offering a scalable method to analyze how patients actually *use* these technologies.

## 3 The infrastructure: from protocol to practice

A powerful algorithm and a validated biomarker are still not a healthcare solution. They must be embedded within a functional infrastructure, a system that spans standardized protocols, usable software, and novel data structures.

This systematic view is championed by Kalia et al. in “Master protocols in vocal biomarker development to reduce variability and advance clinical precision.” This narrative review delivers a powerful, field-defining argument: without *master protocols* for data collection and analysis, the entire field risks stalling in a sea of small, non-comparable, and non-reproducible studies. By drawing on established frameworks from the broader digital-biomarker space (such as V3 and DACIA), the authors provide a roadmap for the standardization needed to build robust, generalizable, and clinically applicable tools. Master protocols, they argue, convert one-off experiments into cumulative evidence, transforming “proof of concept” into regulatory science.

While Kalia et al. provide the 30,000-foot view, Moothedan et al. show what it takes to execute it on the ground. In “The Bridge2AI-Voice application: initial feasibility study of voice data acquisition through mobile health,” they test the very app designed to collect data for the ambitious Bridge2AI project. Their findings are a sobering and crucial reminder: a perfect protocol is useless if the *tool* is unusable. Patients in their feasibility study struggled with task completion and instruction clarity. This paper's contribution is its focus on the often-overlooked work of human-centered design, user experience, and implementation science. It proves that building the app is just as important as building the algorithm. In pragmatic terms, their finding that participants requested assistance in 41% of successfully completed tasks quantifies usability as a scientific variable rather than a *post hoc* concern.

Complementing this work, Anibal et al., in “Voice EHR: introducing multimodal audio data for health,” propose a new data collection approach. This paper challenges the field to move beyond simple, isolated acoustic tasks (like sustained vowels). They introduce the “Voice EHR,” a semi-structured, patient-spoken health record collected through their HEAR application. This method captures not only the *acoustic* properties of the voice, but also the *semantic meaning* of the patient's speech. Using LLMs, they try to show that Voice EHR data is as relevant as, or even superior to, conventional forms. Their innovation reframes voice as both data and narrative, demonstrating that interpretability can emerge from the synergy between voice data and meaning.

## 4 The ecosystem: ethics, education, and enterprise

Technology does not exist in a vacuum, nor is intrinsically neutral. Its success or failure will be determined by the human and techno-social ecosystem that surrounds it. This includes the people we train, the ethical standards we enforce, and the commercial models we build.

The field cannot advance without a new, hybrid workforce. Dorr et al. address this head-on in “Adapting data science competencies by role and purpose: Voice AI.” This paper, developed within the Bridge2AI-Voice Consortium, details an innovative curriculum for training both clinical and technical learners. By using a “persona-based inductive approach,” they are creating the very people who can bridge the gap between the two worlds: the data literate clinician and the clinically fluent data scientist. Their model institutionalizes diversity, equity, inclusion, and accessibility (DEIA) principles at the level of skill formation, linking ethical literacy to technical competence.

As this field develops, it attracts not just academics, but entrepreneurs. Blatter et al. provide a critical analysis of this new commercial sector in “Voice is the New Blood': a discourse analysis of voice AI health-tech start-up websites.” Their study finds a world of promissory and rather futuristic language, but at the cost of risks of transparency gaps, particularly around the training data used to build proprietary algorithms. This is a significant contribution to the ethical discourse, warning of the dangers of “stealth research” and overhyping. Using discourse analysis their paper serves as a critical watchdog, reminding us that public and investor trust is a fragile resource that through overhype and overpromising, once lost, may be difficult to regain. Their analysis exposes a structural asymmetry between innovation speed and proactive accountability, suggesting that regulation must catch rhetoric at the source rather than after deployment.

As a counterpoint and solution, Krautz et al. offer a “Perspective on bridging AI innovation and healthcare: scalable clinical validation methods for voice biomarkers.” Writing from within the commercial sector, they argue that the *only* sustainable path to market is one built on rigor. They advocate for proprietary technology, not as a shield for opacity, but as a tool for deeper analysis (*Musicology AI*). More importantly, they champion large-scale, diverse datasets, strong clinical partnerships, and formal regulatory compliance (for example, medical device certification). This paper provides a compelling commercial and strategic answer to some of the ethical challenges raised by Blatter et al., arguing that for voice AI to succeed as a new business sector, it must first succeed as a rigorous science. Taken together, these two papers define the emerging social contract for voice AI: transparency as currency, certification as legitimacy.

Finally, Malo et al. wrap up this collection with a scoping review of the field's ethical, legal, and social implications (ELSI). The authors delineate a critical boundary between “conventional” digital health risks and “modality-specific” challenges unique to vocal biomarkers. Consequently, the authors reject “exceptionalism” in favor of a “contextualist” framework, asserting that existing bioethical guidance must be rigorously adapted rather than discarded to manage these specific challenges. Their review also uncovers a severe “data divide,” warning that without active correction, the concentration of research in the Global North will reinforce structural health inequities. This work establishes that responsible innovation demands not just technical validation, but a harmonized governance infrastructure that is responsive to the specific inferential power of the human voice.

The unifying paradox of this Research Topic is that openness and protection can coexist. Open datasets accelerate reproducibility but heighten risks of re-identification; proprietary pipelines safeguard quality but risk opacity. Several papers show that the way forward lies in hybrid models: public frameworks combined with auditable, privacy-preserving implementations. This is the field's central intellectual tension and, if managed explicitly, its greatest engine for progress.

## 5 Conclusion: the road ahead

Taken together, these twelve articles provide an, hopefully, clear map of the road ahead. They demonstrate that the future of voice AI in healthcare is not a simple, linear path. It is a complex, multidisciplinary endeavor that demands simultaneous progress on all fronts. The technical power of LLMs, as shown by Shirk et al., is transformative, but it is inert without the usability testing of Moothedan et al. and the standardization advocated by Awan et al. and Nylén. The new data structures proposed by Anibal et al. can only be realized through the rigorous, standardized master protocols that Kalia et al. demand. The foundational clinical validation modeled by Kerwagen et al. and Jenkins et al. serves as the bedrock for the entire enterprise. Finally, this entire scientific and technical stack will either succeed or fail based on its human ecosystem: our ability to train the next generation of hybrid experts (Dorr et al.) and our collective will to build an industry that values transparency (Blatter et al.) and regulatory rigor (Krautz et al.) in context-sensitive approach (Malo et al.) as the core of its business model.

What this collection establishes is not simply a frontier to be discovered, but a discipline to be furthered and governed. The articles here substantially contribute to establishing best practices in the discipline of audiomics, voice AI in healthcare, and vocal biomarkers. Progress may depend on five operational tenets: (1) adopt master protocols aligned with verification, analytical, and clinical validation stages; (2) treat usability metrics as primary outcomes; (3) require public model and data cards describing linguistic and demographic coverage; (4) integrate DEIA training into every technical curriculum; and (5) pursue early regulatory alignment rather than retrospective compliance. This Research Topic does not mark the arrival of voice AI in healthcare. It marks the end of the beginning. It provides a comprehensive, clear-eyed, and multidisciplinary guide for the hard work that lies ahead: the work of building a future where this remarkable technology is not just innovative, but effective, equitable, responsible, and worthy of our trust.
